# Protective Effect of DNA Vaccine Encoding *Pseudomonas* Exotoxin A and PcrV against Acute Pulmonary *P. aeruginosa* Infection

**DOI:** 10.1371/journal.pone.0096609

**Published:** 2014-05-01

**Authors:** Mingzi Jiang, Jing Yao, Ganzhu Feng

**Affiliations:** Department of Respiratory, the Second Affiliated Hospital of Nanjing Medical University, Nanjing, Jiangsu Province, China; The Scripps Research Institute and Sorrento Therapeutics, Inc., United States of America

## Abstract

Infections with *Pseudomonas aeruginosa* have been a long-standing challenge for clinical therapy because of complex pathogenesis and resistance to antibiotics, thus attaching importance to explore effective vaccines for prevention and treatment. In the present study, we constructed a novel DNA vaccine by inserting mutated gene *toxA_m_* encoding *Pseudomonas* Exotoxin A and gene *pcrV* encoding tip protein of the type III secretion system into respective sites of a eukaryotic plasmid pIRES, named pIRES-*toxA_m_-pcrV*, and next evaluated the efficacy of the vaccine in murine acute *Pseudomonas* pneumonia models. Compared to DNA vaccines encoding single antigen, mice vaccinated with pIRES-*toxA_m_-pcrV* elicited higher levels of antigen-specific serum immunoglobulin G (IgG), enhanced splenic cell proliferation and cytokine secretion in response to *Pseudomonas aeruginosa* antigens, additionally PAO1 challenge in mice airway resulted in reduced bacteria burden and milder pathologic changes in lungs. Besides, it was observed that immunogenicity and protection could be promoted by the CpG ODN 1826 adjuvant. Taken together, it’s revealed that recombinant DNA vaccine pIRES*-toxA_m_-pcrV* was a potential candidate for immunotherapy of *Pseudomonas aeruginosa* infection and the CpG ODN 1826 a potent stimulatory adjuvant for DNA vaccination.

## Introduction

*Pseudomonas aeruginosa* is an opportunistic gram-negative pathogen causing acute or chronic infections in patients with compromised immunity, burned injury and cystic fibrosis. Characterized by high incidence, severe symptoms and increasing drug resistance, *P. aeruginosa* infections have been difficult to prevent or cure, thereby it is urgent to explore new therapeutic options. As understanding of *P. aeruginosa* pathogenesis and virulence factors grows deeper, more potential immunogens that could be used for *Pseudomonas* vaccine have been identified [1]–[4]. Over recent decade, DNA vaccines have proved to be effective in animal models and extensively put into Phase I-III clinical trials in humans [5], [6]. It’s revealed referring to past researches that DNA vaccines have distinctive advantages on preparation, delivery, stability, administration and safety over other conventional vaccines [7], [8] and offer the possibility to simultaneously target different antigens, thus being considered as an attractive approach for antigen-specific immunotherapy [9], [10]. Although no effective DNA vaccines against *P. aeruginosa* have been available clinically [2], [11], the genetic immunization targeting various antigens, such as Outer Membrane Protein, Flagella, exotoxin A, have been reported to be immunogenic and protective in animal models [12]–[17].

Acute *P.aeruginosa* infections, such as nosocomial pneumonia and infection for immunocompromised patient, are invasive and cytotoxic, frequently resulting in substantial tissue damage, systemic spread, sepsis even death. Corresponding mechanism comprises surface factors of *P. aeruginosa* contributing to bacterial adherence and colonization, while different secreted proteins decisive in dissemination and tissue damage [1], [18]. *P. aeruginosa* has a large complement of secreted proteins and five secretion systems, among which the Type II and Type III secretion system (T2SS and T3SS) have been proved to be associated with highest morbidity during acute infections [19]–[22]. Previous studies demonstrate that the T3SS acts rapidly to help bacteria evade phagocytosis, while the T2SS disturbs the clearance of pathogen at a slow rate. Since these two secretion systems may be viewed together as comprising a fail-safe system for defense against pathogens and are both integral to pathogenesis, it’s suggested that a specific toxic secretion product of the T2SS needs to be included in a vaccine designed to target secretions [23].

The T2SS secretes Exotoxin A, proteases, phospholipase H and lipolytic enzymes [24]. As an ADP-ribosyl transferase inhibiting elongation factor-2 (EF-2), Exotoxin A blocks protein synthesis thus leading to cell death and has also been shown to depress host response to infection [25]–[28]. Given its toxicity, modifications of naive Exotoxin A were investigated in order to obtain non-toxic but suitable immunogenic protein. A novel DNA vaccine encoding truncated Exotoxin A gene has been proved to be able to express Pseudomonas Exotoxin A (PE) protein in vivo, induced specific immune response, and provided sufficient protective immunity that safeguarded mice from the injection of lethal dosage of PE toxin [16]. Investigations on fusion protein vaccines, e.g. PE-OprF-OprI [29], PE-alginate [30], LPS-PE [31] and PE-flagellin [32] revealed practical methods on non-toxic alteration of Exotoxin A. These studies proposed Pseudomonas Exotoxin A important vaccine candidate.

The T3SS allows the bacterium to directly inject toxins into host cells, thus significantly associated with tissue damages. Between 75–90% of isolates from patients with *P. aeruginosa*-mediated acute respiratory infections have genes for T3SS [33], [34]. PcrV is a component of translocation apparatus of T3SS, proved to participate in the regulation of translocator pore assembly and effectors delivery. Moreover, like ortholog LcrV for *Yersinia*, IpaD for *Shigella flexneri*, BipD for *Burkholderia pseudomallei*, and SipD for *Salmonella spp.*, PcrV is an important protective antigen against T3SS-mediated *P. aeruginosa* infection [35]. Previous studies have revealed that either active or passive anti-PcrV treatment elicited potent immunogenicity and provided effective protection for animal models challenged with *P. aeruginosa*, thus indicating PcrV practicable candidate as immunogens [36]–[42].

Unmethylated cytosine-phosphate-guanosine(CpG) oligodeoxynucleotides (ODNs) are recognized by Toll-like receptor 9 (TLR9) in of dendritic cells and B cells to induce cytokines, activate natural killer cells and elicit T-cell responses. It had been revealed that CpG ODNs could act as a potent adjuvant for treating infectious diseases, cancers, and allergies. The specific CpG ODN 1826, a well-defined B class CpG DNA with strong immunostimulatory properties in mice, can promote vaccine efficacy against infectious disease or cancer and prevents infection from bacteria in murine model [43]–[48].

In the present study, we generated a novel DNA vaccine simultaneously containing genes encoding Exotoxin A and PcrV and have investigated its immunogenicity and protective potential against *P. aeruginosa* challenge in mice. Besides that, evaluations on the potency of the CpG ODN 1826 as immunoadjuvant to enhance immunity and protection were carried out in order to explore a potential approach to promote immunization efficacy.

## Materials and Methods

This study was approved by the Institutional Ethical Guidelines for Animal Experiments of Nanjing Medical University (permit number: IACUC, Institutional Animal Care and Use Committees, 2010257). All surgery was performed under sodium pentobarbital anesthesia, and all efforts were made to minimize suffering.

### Construction of DNA Vaccines

The eukaryotic expression plasmid, pIRES vector, was used in this study. This plasmid contains an immediate early promoter of cytomegalovirus (CMV promoter), an intron (IVS), MCS A, internal ribosome entry site (IRES), and MCS B followed by simian virus (SV40) polyadenylation signals to ensure efficient expression of two target protein in eukaryotic host cells.

Genomic DNA of *P. aeruginosa* strain PAO1, a reference strain used for Pseudomonas genetics and functional analyses, was prepared as described previously [49]. The *toxA* and *pcrV* genes were PCR-amplified from genomic DNA of PAO1 [17], [50], respectively labeled by a Human influenza hemagglutinin (HA)-tag sequence and a His-tag sequence. Gene *toxA*-HA was mutated following previous methods [51], namely *toxA*_m_-HA. Then the gene fragment *toxA*_m_-HA was inserted in MCS A (NheI-EcoRI) and *pcrV*-His in MCS B (XbaI-SalI), to construct recombinant DNA vaccines pIRES-*toxA*_m_, pIRES-*pcrV* and pIRES-*toxA*_m_-*pcrV*. All plasmids were confirmed by DNA sequencing.

### Expression of toxA^m^ and pcrV Genes in vitro

The recombinant plasmids were transient transfected into Human embryonic kidney (HEK-293) cells using lipofectamine 2000 (Life Technologies, California, USA ). After cultured for 48 hours, cell lysates harvested were analyzed by western bolt using anti-HA monoclonal antibodies and anti-His monoclonal antibodies (Genscript, Nanjing, China).

### Recombinant Proteins

Recombinant protein PcrV and Pseudomonas Exotoxin A (PE) were prepared and purified as previously described [36], [52].

### Preparation of the CpG ODN 1826

Nuclease-resistant phosphorothioate-modified ODN 1826 (5′- TCCATGACGTTCCTGACGTT-3′) was synthesized by Invitrogen (Shanghai) The CpG ODN 1826 (5′-TCCATGACGTTCCTGACGTT-3′) were synthesized with a nuclease-resistant phosphorothioate backbone (Sangong, Shanghai China) and dissolved in endotoxin-free phosphate-buffered saline (PBS) as described before [47].

### Mice

Female BALB/c mice (6–8 weeks old) were purchased from the central animal laboratory of Yangzhou University (Yangzhou, China) and kept in specific pathogen-free condition (eight in each group). This study was carried out in strict accordance with the recommendations in the the Regulations for the Administration of Affairs Concerning Experimental Animals approved by the Institutional Ethical Guidelines for Animal Experiments of Nanjing Medical University (permit number: IACUC 2010257). All surgery was performed under sodium pentobarbital anesthesia, and all efforts were made to minimize suffering.

### Intramuscular Immunization of Mice

After anesthetized by vaporized isoflurane, mice were injected in quadriceps muscles with the plasmid solution (1 µg/µl saline, 50 µl/muscle) respectively containing 100 µg of pIRES-*toxA_m_*, pIRES-*pcrV*, pIRES-*toxA_m_-pcrV*. For evaluation on adjuvant, another three groups of mice were respectively injected with 100 µg of each plasmid plus 15 µg of CpG ODN 1826. The mice of control groups were treated with the pIRES (blank plasmid) and PBS, respectively. Mice were administrated for a total of three times at 2-week intervals, serum was prepared 1 week after each administration and stored at −70°C until analysis.

### The Proliferation and Cytokine Responses of Spleen Cells after Restimulation with Antigens

One week after the final immunization the mice were euthanized and their spleens removed under aseptic conditions. Single-cell suspensions were prepared from the spleens according to standard procedures. For the proliferation response, splenic cells were cultured at 37°C with 5% CO_2_ in a 96-well flat-bottom plate at a concentration of 4×10^5^ viable cells/well and incubated with the following different stimulants for 72 hours: 10 µg/ml of recombinant PcrV, Exotoxin A prepared before and the control Concanavalin A. The proliferation response of splenic cells were determined by 3-(4, 5-dimethylthiazol-2-yl)-2,5-diphenyltetrazolium bromide (MTT) method [53].

For cytokines detection, the splenic cells were plated at 2×10^6^ cells/well in flat-bottom well plates and stimulated with recombinant PcrV or Pseudomonas Exotoxin A (PE) for 72 hours. Supernatants were collected afterwards and assayed for cytokine production. Levels of IFN-γ, IL-12, IL-4 and IL-10 were measured by antigen-capture ELISA using mouse IFN-γ, IL-12, IL-4 and IL-10 detection kit (eBioscience, San Diego, CA).

### Antigen-specific Antibodies Detection

Anti-PcrV IgG and anti-ExotoxinA IgG in serum samples were measured by Enzyme Linked Immunosorbent Assay (ELISA). Recombinant protein PcrV and Pseudomonas Exotoxin A (PE) prepared before were used as coated antigens.

### Respiratory Challenge with *Pseudomonas Aeruginosa*

The *Pseudomonas aeruginosa* strain PAO1, a reference strain for *Pseudomonas* genetics and functional analysis, was used for the challenge procedure. PAO1 was cultured in Terrific broth (Sigma, USA) at 37°C overnight, then the bacterial culture was centrifuged at 3000×g for 10 minutes, the pellet washed twice in PBS and resuspended in PBS at an optical density of 1.0 at 550nm.

Two weeks after final immunization, each mouse was anesthetized and inoculated intranasally with 5×10^7^ CFU of PAO1 [21], [54]. 72 hours later, infected mice were euthanized, their lungs were removed and homogenized in 4 ml PBS under sterile conditions. Serial 10-fold dilutions of lung homogenates were plated on Terrific broth, and incubated at 37°C for 24 h. The number of colonies was enumerated manually in a double-blinded manner. Besides, the lung samples of mice were subjected to hematoxylin-eosin (HE) staining for observation on histological changes of lung inflammation and damage. A quantitative morphometric analysis of intra-alveolar exudate, interstitial edema, alveolar hemorrhage, and inflammatory cell infiltration was performed, as has been described previously [55]. Each item was scored 0–3 (0 = normal; 1 = mild; 2 = moderate; 3 = severe) and the score for each animal was calculated by dividing the total score for the number of sections observed.

### Statistics

One-way ANOVA analysis of variance was performed to analyze differences in means between the experimental groups (SPSS Statistics 18.0). P values of <0.05 were considered significant.

## Results

### Plasmid Construction and Transient Expression of *toxA* and *pcrV* Gene *in vitro*

**Figure 1:**
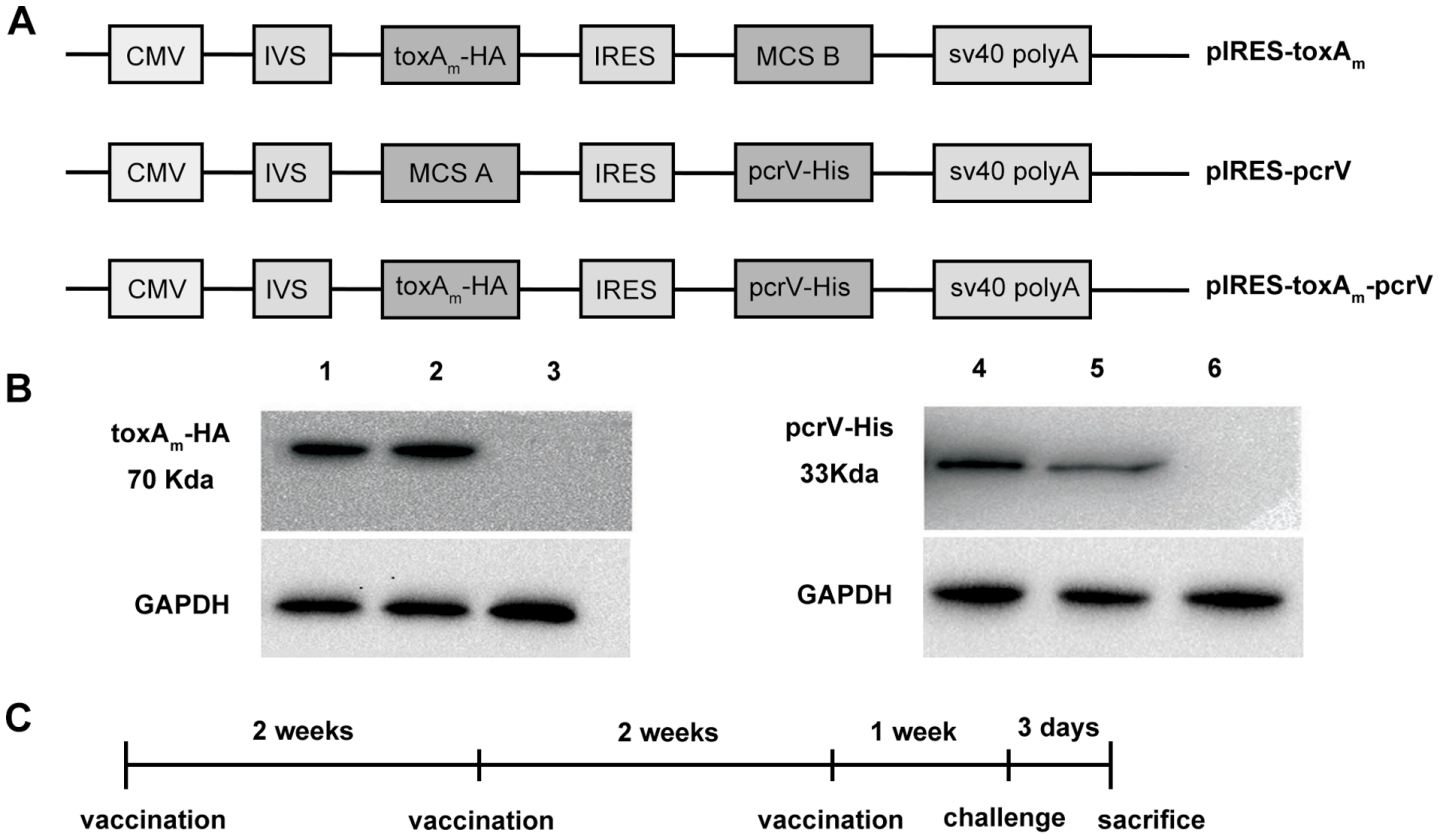
Construction and identification of recombinant DNA vaccines. **A)** Schematic construction of recombinant plasmid pIRES*-toxA*_m_, pIRES*-pcrV*, and pIRES*-toxA*_m_ *-pcrV*. The *toxA*_m_ gene and *pcrV* gene were respectively tagged by HA tag and His tag. **B)** The expression of *pcrV*-His gene and *toxA*_m_-HA gene in HEK-293 cells by western blot using anti-His monoclonal antibody and anti-HA monoclonal antibody respectively. Channel 1 to 6 represents plasmids transfected: 1 pIRES-*toxA_m_*, 2 pIRES-*toxA_m_-pcrV*, 3 pIRES, 4 pIRES-*pcrV*, 5 pIRES*-toxA_m_-pcrV*, 6 pIRES. **C)** The schedule of immunization and PAO1 challenge.

According to the schematic diagram in Figure 1 A, the mutated *toxA* gene for P aeruginosa ExotoxinA and the *pcrV* gene for T3SS protein PcrV were inserted into eukaryotic expression vector pIRES, respectively adding HA tag and His tag to each gene for expression detection. As depicted in Figure 1 B, the expressions of Exotoxin A_m_-HA and PcrV-His protein in HEK-293 cells were identified by western blot, determining the ability of recombinant DNA vaccines to express target proteins in eukaryotic expression system.

### Humoral Immune Response to DNA Vaccines

**Figure 2:**
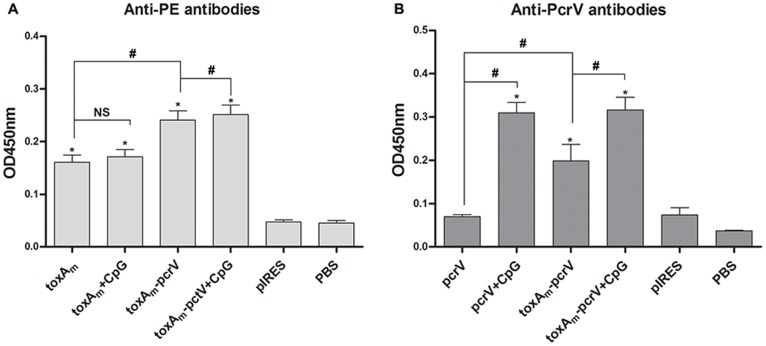
Characterization of humoral immune response elicited by DNA vaccination. Sera from groups of mice immunized intramuscularly with DNA vaccines with and without the CpG ODN 1826 were analyzed for the presence of antigen-specific antibodies by ELISA. **A)** Comparison of anti-PE specific antibodies. **B)** Comparison of anti-PcrV specific antibodies. Bar, mean and SEM from 3 independent experiments, each using at least three mice per group (n = 15 for one vaccine group); # P<0.05 between the indicated pairs, NS no significance in statistics, * P<0.05 versus PBS vaccinated group.

Antigen-specific antibodies in serum from immunized mice were tested by ELISA using PE and PcrV as coating antigens. As shown in Figure 2, one week after final administration, all the three DNA vaccines elicited sufficient amounts of antigen-specific antibodies compared to the PBS inoculated control group (P<0.05). Meanwhile, the level of antibodies in pIRES-*toxA_m_-pcrV* group was significantly higher than pIRES-*toxA_m_* and pIRES-*pcrV* group. Besides, increased productions of specific antibodies were observed in serum of mice inoculated with DNA vaccine plus CpG ODN 1826 versus the corresponding vaccination without adjuvant group (P<0.05).

### T cell Immune Response to DNA Vaccines

**Figure 3:**
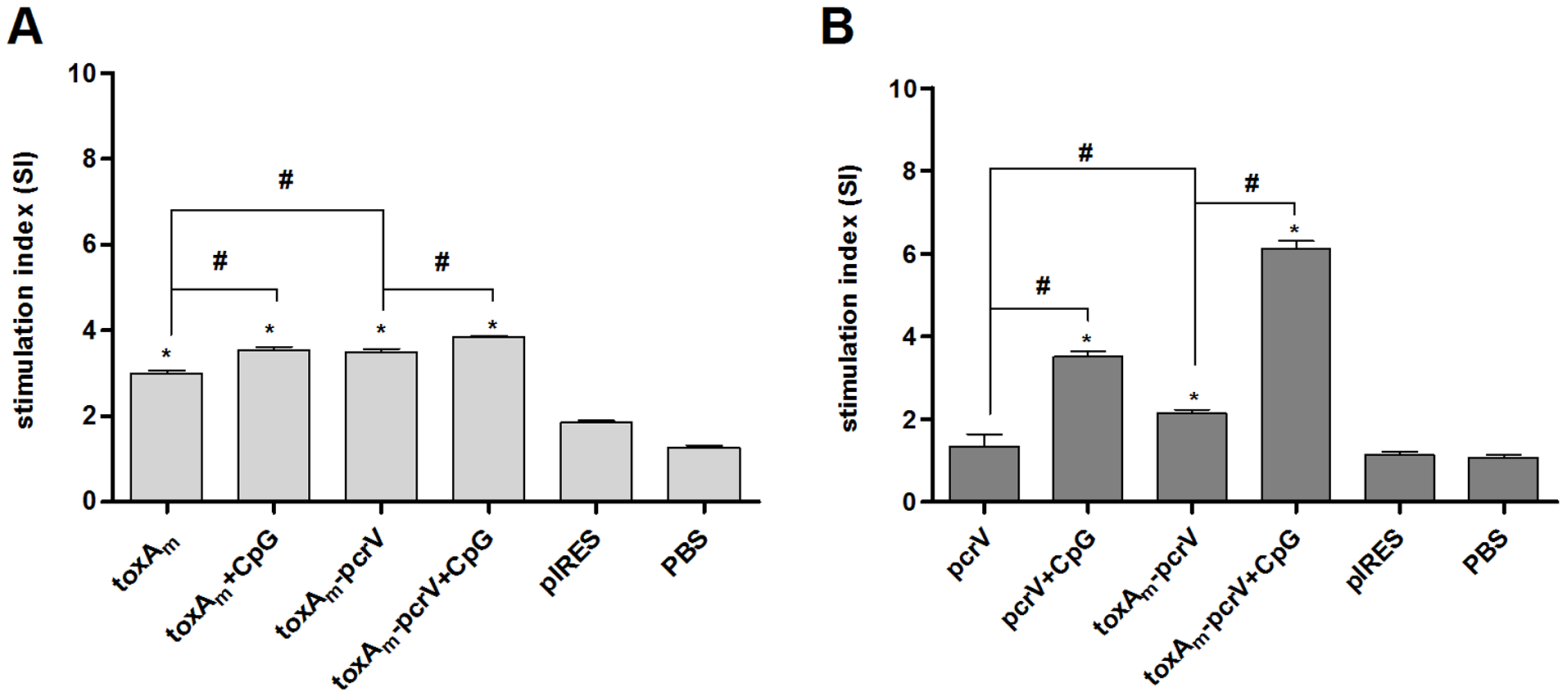
Proliferation of splenic cells from mice immunized with DNA vaccines. One week after the last administration, splenic cells of mice were collected and cultured respectively with **A)** Pseudomonas Exotoxin A and **B)** recombinant PcrV for 72 hours, then the stimulation index (SI) of splenic cells within different groups was calculated to determine the proliferation activity. Bar, mean and SD from 2–4 independent experiments, each using at least three mice per group (n = 15 for one vaccine group); # P<0.05 between the indicated pairs, *P<0.05 versus PBS vaccinated group.

Examinations of the proliferative response and cytokine secretion profile in vitro were carried out by re-stimulating splenic cells from immunized mice respectively with 10 µg/ml of recombinant PE and PcrV as antigen. Significant SI (stimulation index) in each DNA vaccinated group was indicated in Figure 3 (P<0.05 versus PBS control). Splenic cells from pIRES-*toxA_m_-pcrV* treated group achieved greater proliferation than pIRES-*toxA_m_* and pIRES-*pcrV*, either stimulated with PE or PcrV (P<0.05 between group pairs). Meanwhile, stimulation index significantly increased in DNA vaccine plus CpG ODN 1826 groups.

**Figure 4:**
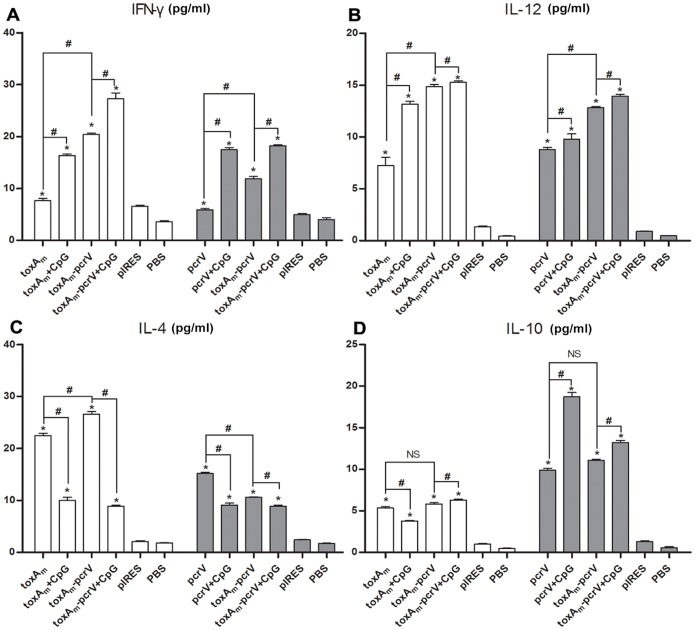
Concentration of cytokines in the supernatants of antigen-stimulated splenic cells from immunized mice. One week after the last vaccination, splenic cells from mice were respectively stimulated by Pseudomonas Exotoxin A and recombinant PcrV for 72 hours. Afterwards cell supernatants were collected to examine the levels of cytokines **A)** IFN-γ **B)** IL-12 **C)** IL-4 and **D)** IL-10 by ELISA. Bar, mean and SD from 2–4 independent experiments, each using at least three mice per group (n = 15 for one vaccine group); # P<0.05 between the indicated pairs, *P<0.05 versus PBS vaccinated group.

The contents of cytokines secreted by antigen-stimulated splenic cells were detected by ELISA. As shown in Figure 4, splenic cells in DNA immunized mice exhibited sufficient ability to induce cytokines compared to PBS and pIRES control groups. When respectively compared to single-gene-encoding DNA vaccine, higher yields of IFN-γ, IL-12 were achieved in pIRES-*toxA_m_-pcrV* group, meanwhile the amount of IL-4 production increased when splenic cells were stimulated by PE antigen whereas decreased when stimulated by PcrV antigen, and the secretion of IL-10 did not change significantly. In groups of mice co-immunized with the CpG ODN 1826, elevated secretion of IFN-γ and IL-12 but reduced IL-4 production occurred in each DNA vaccine group. As to IL-10 secretion, interestingly, the CpG adjuvant caused slight decline for pIRES-*toxA_m_* vaccine but remarkable augment for pIRES-*pcrV* vaccine, and a moderate rise for pIRES-*toxA_m_-pcrV* vaccine in both PE-antigen and PcrV-antigen stimulation.

### Protective Effect on Vaccinated Mice Against *Pseudomonas Aeruginosa* Challenge

In murine model of acute *P. aeruginosa* infection, histological observation, bacteria count, injury score of mice lungs were carried out to evaluate the protective efficacy of the novel DNA vaccine, results shown respectively in Figure A, B and C. The pIRES and PBS control groups displayed severe interstitial pneumonia including serious damage of alveolar structure, large infiltration of inflammatory cells and amounts of bacteria colonization, while mice immunized with DNA vaccines displayed moderate damage with better lung structure, relatively fewer neutrophil infiltration and bacterial invasion. Among them, pneumonia in pIRES*-toxA_m_-pcrV* immunized mice displayed lightest pathologic changes and least bacterial burden in lung tissues. Additionally, in the groups of vaccination plus CpG ODN 1826 improved structural integrity, decreased inflammatory infiltration and enhanced elimination of bacteria were observed.

## Discussion

*Pseudomonas aeruginosa* has been noted for its environmental versatility, ability to cause disease in particular susceptible individuals, and its resistance to antibiotics. The multifactorial and complex pathogenesis of *P. aeruginosa* infection include attachment and colonization, local infection, and finally bloodstream dissemination and severe systemic damage [18]. It’s revealed that *P. aeruginosa* possesses various protein secretion systems to invade and damage host cells, of which the type II secretion system (T2SS) and type III secretion system (T3SS) secrete the majority of known toxins [23], [56], [57]. As an important cytotoxic extracellular protein, Exotoxin A also acquires effective immunogenicity, making it potential candidate for *Pseudomonas* vaccine [58]–[60]. PcrV is a key component of T3SS, regulating the assembly of virulence effector-delivery structure in eukaryotic cell membrane, besides, efficacy of anti-PcrV immunotherapy in animal of *P. aeruginosa* infection suggested the immune-protective feasibility of PcrV [36], [61]. In the present study, intending to explore the genetic immunization simultaneously targeting Exotoxin A and PcrV, we constructed a recombinant DNA vaccine pIRES*-toxA_m_-pcrV,* which contained the Glu-553-mutated *toxA* gene and the *pcrV* gene, and evaluated the immunogenicity and protective capacity in mice.

**Figure 5:**
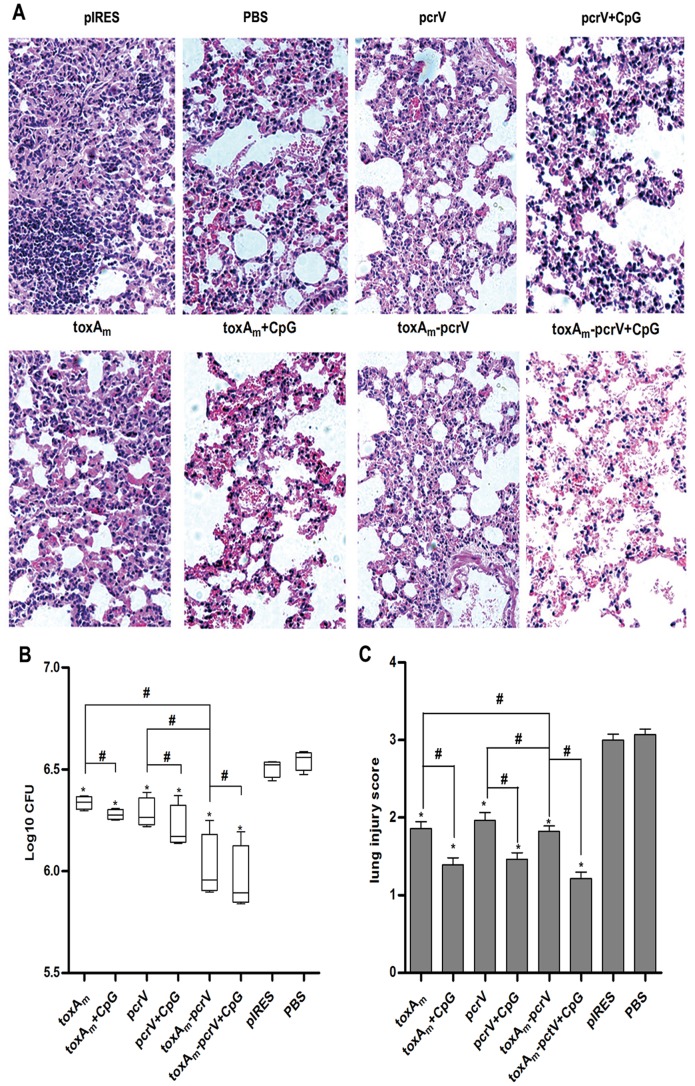
Protective effects against *PAO1* challenge in the lungs of immunized mice. One week after the final immunization, mice were intratracheally inoculated with PAO1 and three days later, lungs were collected to analyze the bacterial and pathologic changes. **A)** Histopathological observation and **B)** Area of inflammatory cells infiltration of the lungs of pathogen-challenged mice (HE stained, magnification ×200). **C)** scores for lung injury of infected mice. Homogenates of lungs from sacrificed mice were prepared, inoculated in Terrific broth at 37°C and bacterial burdens were counted after 24 hours. Bar, mean and SEM from 3 independent experiments, each using at least three mice per group (n = 15 for one vaccine group). # P<0.05 between the indicated pairs, *P<0.05 versus PBS vaccinated group.

Expression of Exotoxin A and PcrV in HEK-293 cells confirmed the ability of pIRES-*toxA_m_-pcrV* to function in eukaryotic systems. DNA vaccines were inoculated in mice, following detection of humoral and cellular immunity. Compared to pIRES-*toxA_m_* or pIRES-*pcrV*, higher yields of both anti-PE and anti-PcrV antibodies were generated in pIRES-*toxAm-pcrV* group, indicating greater capacity to induce humoral immunity as shown in Figure 2 and 3. Through measurements on proliferation and cytokine secretion of antigen-stimulated splenic cells from vaccinated mice, it was revealed that pIRES-*toxA_m_-pcrV* possessed stronger potency to evoke splenic cell proliferation than either pIRES-*toxA_m_* or pIRES-*pcrV*. The cytokine secretion of splenic cells indicated that pIRES-*toxA_m_-pcrV* could induce efficient Th1-type cytokines by both antigen stimulation and acquire greater efficacy than either pIRES-*toxA_m_* or pIRES-*pcrV*. As to Th2-type cytokines, pIRES-*toxA_m_-pcrV* stimulated by PE antigen generated more IL-4 than pIRES-*toxA_m_* but less than pIRES-*pcrV* when stimulated by PcrV-antigen, meanwhile, no significant change occurred in IL-10 production by both antigen stimulation. These results implied that the DNA vaccines containing mutated *toxA* gene and *pcrV* gene were effective on inducing immune response. Following the immunogenicity assessments, protection capacities of these DNA vaccines were evaluated in murine model of acute *P.aeruginosa* pneumonia. The comparisons of pathogen clearance, inflammatory cell infiltration and lung injury score assessments were consistent with previous results, which were depicted in Figure 5 showing that lung destruction lightened while pathogen elimination increased in DNA vaccinated groups compared to control groups, besides, among the three DNA vaccines pIRES-*toxA_m_-pcrV* provided better protection against infection than either pIRES-*toxA_m_* or pIRES-*pcrV*. To sum up, recombinant DNA plasmid co-encoding *toxA_m_* and *pcrV* is more effective in eliciting humoral and cellular immunogenicity as well as protecting mice against *P.aeruginosa* challenge.

Synthetic oligodeoxynucleotides (ODN) that contain unmethylated motifs (CpG ODN) could trigger cells expressing Toll-like receptor 9 to improve the function of professional antigen-presenting cells and boost the generation of humoral and cellular vaccine-specific immune responses. In the present study, we used the CpG ODN 1826 (a B-class CpG ODN specific for mouse TLR9) as adjuvant to the DNA vaccines to investigate its potential in enhancing immunity and protection. According to our results, the adjuvant could effectively promote the humoral immune responses induced by DNA vaccination, especially for the pIRES-*toxA_m_-pcrV*. Among the results, it seemed that pcrV vaccine efficacy could be elevated strikingly by the CpG ODN 1826, similar results found in LcrV vaccine study of *Yersinia pestis* [62]. In cellular immunity, the CpG ODN 1826 successfully elevated antigen-stimulation index of splenic cells, particularly in pIRES-*toxA_m_-pcrV* group. Furthermore, the CpG adjuvant enhanced the Th1/Th2 shift cytokine secretion, with increased production of IFN-γ and IL-12 while decreased IL-4 generation, indicating a better Th1-bias tendency than no-adjuvant groups. Interestingly, with regards to IL-10, a slight decline in pIRES-*toxA_m_* but significant augment in pIRES-*pcrV* caused by CpG ODN 1826 was observed. Referring to past researches, IL-10 could be generated by various types of T helper cells, collaboratively promoting antigen presentation, inflammation and immunoregulation, and CpG ODNs were able to up-regulate IL-10 levels as well, therefore, the present result that production of IL-10 augmented in PcrV-stimulation group seemed reasonable [63], [64]; however, Exotoxin A acquired a suppressive effect on IL-10, as a result, the rise of IL-10 observed in PcrV stimulation might be diminished by Exotoxin A, consequently displaying a moderate augment on IL-10 of pIRES-*toxA_m_-pcrV* [65]. Yet, the CpG ODN 1826 adjuvant could be considered effective on enhancing the Th1-shift immunity of DNA vaccines, consistent with past results reported [45]. Regarding to protective efficacy, less bacteria colonization and lightened lung tissue injury were observed as displayed in Figure 5. To sum up, the CpG ODN 1826 achieved potent capacity to promote immunogenicity and protection of DNA vaccination.

Persistent *P. aeruginosa* infection is the major cause of chronic inflammation and various factors affect efficiency of vaccines [66]. More researches on optimizing animal model of chronic *P. aeruginosa* infection are required as well as proper adjuvants are in urgent need to strengthen potency of DNA vaccines. In conclusion, the present study directly demonstrates DNA vaccine encoding *pcrV* and attenuated-*toxA* gene a potential candidate for immunotherapy against acute *P. aeruginosa* infection and CpG ODN an effective immune-adjuvant, therefore provided novel strategy for prevention and treatment of *P. aeruginosa.* Given complexity of pathogenesis and genetics, there’s still a tough-long way on exploration of an applicable vaccine against *Pseudomonas aeruginosa* until practical application in clinical treatment.
